# Translation and validation of the Chinese ABCD risk questionnaire to evaluate adults’ awareness and knowledge of the risks of cardiovascular diseases

**DOI:** 10.1186/s12889-022-14101-z

**Published:** 2022-09-03

**Authors:** Yan Liu, Wei Yu, Mei Zhou, Fang Li, Farong Liao, Zhengyu Dong, Hairong Wang, Jiaqing Chen, Lingling Gao

**Affiliations:** 1 Nursing Department, Affiliated Hospital of Guilin Medical University, Guangxi, China; 2 School of Nursing, Sun Yat-sen University, Guangzhou, China; 3 Cardiovascular Medicine Ward, Affiliated Hospital of Guilin Medical University, Guangxi, China; 4 Zhongshan School of Medicine, Sun Yat-sen University, Guangzhou, China; 5 Rhode Island Hospital, affiliated with the Warren Alpert Medical School of Brown University, Providence RI, USA

**Keywords:** Cardiovascular disease, Health beliefs, Risk perception, Translation

## Abstract

**Background:**

Assessment of health beliefs and risk perception is a critical means to prevent coronary heart disease, but there are few such studies on assessment in the Chinese population. Given the demonstrated value and widespread use of the Attitudes and Beliefs about Cardiovascular Disease Risk Questionnaire (ABCD), this study was designed to translate it into Chinese, and to evaluate its reliability and validity in a Chinese population.

**Methods:**

The Chinese version of the ABCD was created using the Beaton translation model, which included forward and backward translation. The reliability and construct validity of the Chinese ABCD were examined in a sample of 353 adults who participated in the public welfare projects of the Chinese National Center for Cardiovascular Diseases in Guilin city, Guangxi. Exploratory factor analysis (EFA) and confirmatory factor analysis (CFA) were performed to examine the factor structure of the Chinse ABCD. The internal consistency of the questionnaire was assessed using Cronbach’s α and corrected item-total correlations.

**Results:**

We deleted item 7 in the knowledge dimension of the Chinese ABCD and added two items about smoking and sleep knowledge, while retaining 25 of the original items, so that it finally included 27 items. The correlations were .20–.90; the correlations between each item and the total score of the ABCD were .34–.86; and the item-level Content Validity Index (I-CVI) was .86–1.00. The results of the EFA showed that all items were close to .40, and the cumulative variance contribution rate was 63.88%. The model fit was acceptable (χ^2^ = 698.79, *df* = 243, χ^2^/*df* = 2.87, *P* < 0.001, SRMR = 0.06, RMSEA = 0.05, CFI = 0.96, and TLI = 0.94) according to the CFA. The Cronbach’ s α of the entire questionnaire was .86, and the α of each of dimension was .65, .90, .88, and .78. The split-half reliability of the entire the ABCD was .67, and the test-retest reliability was .97 (*P* < 0.05). The questionnaire had good reliability and validity and was associated with sociodemographic and health-related characteristics (smoking and Body Mass Index).

**Conclusion:**

The Chinese version of the ABCD has good reliability and validity, and provides a reliable assessment tool for measuring public health beliefs about the risk of cardiovascular disease, promoting the primary prevention of coronary heart disease.

**Supplementary Information:**

The online version contains supplementary material available at 10.1186/s12889-022-14101-z.

## Introduction

Cardiovascular diseases (CVDs) are the leading cause of death and disability in the world, mainly because of ischemic heart disease and stroke [1]. According to the latest Global Burden of Cardiovascular Diseases study, the number of patients worldwide with CVDs reached 523 million in 2019, and the morbidity due to CVDs was 330 million in China. Furthermore, the highest rates of morbidity and mortality from CVDs are in China [2], which is partly related to the increase in the elderly population of China. Because long-term, unhealthy lifestyles exacerbate the risk of CVDs in the elderly, the Chinese Guidelines on Healthy Lifestyle to Prevent Cardio-metabolic Diseases make some recommendations to reduce risk factors, such as, to stop smoking, to eat a rational diet, and to engage in physical activity and other healthy lifestyle habits. Altering bad habits and maintaining a healthy lifestyle is important to prevent CVDs, which are affected by one’s health beliefs. It is well known that health beliefs affect one’s perceptions and health knowledge of behavioral risks [3, 4]. There is evidence that individuals who have health knowledge will engage in healthier behaviors to reduce the incidence of CVDs [5].

Therefore, effective and reliable assessments of individuals’ knowledge and perceptions of risks are essential. In 2015, Liu et al. developed a Chinese version of a health-belief scale for diabetic patients about the prevention of CVDs [6], but it was not for the general population. At present, the Attitudes and Beliefs about Cardiovascular Disease Risk Questionnaire (ABCD), developed by the British National Health Service Program to measure the general population’s perceptions and knowledge of the risks of CVDs, is widely used abroad [7–9]. However, the ABCD has not been translated to Chinese and validated in a Chinese sample. Hence, this study’s aims were to translate the ABCD to Chinese and to evaluate its psychometric performance in a Chinese sample using classical test theory. In addition, the Chinese version of the questionnaire was applied to the cognition and assessment of CVD risk in a population in a cardiovascular disease screening program.

## Methods

### Sample and procedures

A convenience sample of persons who attended a CVD screening program was recruited for the study from an outpatient department of the Affiliated Hospital of Guilin Medical University from October 2021 to January 2022 in Guilin, Guangxi province, China. The inclusion criteria were: being a permanent resident of Guilin for over 6 months, age 35 years or older, and not being diagnosed with a mental or cognitive disorder.

The sample size was determined based on the general rule that the sample should contain 5–10 participants for each item to be analyzed by factor analysis. Given that the English ABCD questionnaire has 26 items, and assuming a 20% rate of invalid questionnaires, the calculated sample size was 325 cases, but it was determined that the sample size should be 374 cases.

### Measures

#### The original ABCD

The ABCD is a self-assessment tool to evaluate of an individual’s health knowledge, perceived risks, and benefits, which was developed in 2017 by Woringer et al. [7], based on the Health Belief Model and the Transtheoretical Model. It consists of 26 items that measure four dimensions, including CVD knowledge, perception of risks, perception of benefits, and healthy eating intentions. The knowledge dimension is measured using dichotomous response options (yes/no questions), and the other three dimensions are measured using a 4-point Likert scale, with responses ranging from 1 = “completely disagree” to 4 = “completely agree.” The ABCD’s total score ranges from 18 to 80 points. The higher the score, the higher the perceived risk of preventing CVD. It is currently used to assess the perceived risk of CVD in England’s health-examination population, the Hungarian community population [8], and Dutch adults [9, 10].

### Translation and adaptation of the Chinese ABCD

To ensure the quality of the research methodology, the questionnaire was evaluated according to the contents of the COnsensus-based Standards for the selection of health status Measurement INstruments (COSMIN) checklist [11], and the study’s report was adhered to the Strengthening the Reporting of Observational Studies in Epidemiology (STROBE) [12]. After obtaining the consent and authorization of the original author of the ABCD, a research group was established to perform a Chinese translation of it using the Beaton translation model [13, 14]. First, forward translation of the ABCD was performed independently by two experts who had experience translating medical questionnaires abroad for 2 years. A comprehensive analysis of the two translations was conducted to select the most appropriate question content for the Chinese version, and version 1 was created. Second, the Chinese version was back-translated to English independently by language professionals in Sun Yat-Sen University and a doctor of cardiovascular medicine in the United States who lived and worked there for over 20 years. After discussion about and analysis of the two translated versions, a comprehensive translated version was created. Third, the second Chinese version (version 2) was revised based on the review and discussion of it by the members of an opinion group. Next, the field Chinese version was sent to an expert committee of who reviewed the translation methodology to make cultural adjustments for Chinese populations. Finally, the 40 patients who met the standards of admission to the study were selected to complete the Chinese version in order to evaluate its reliability and validity. After modifying the wording of the individual items of the questionnaire, the expert committee reviewed and evaluated it again, and the final Chinese version of the questionnaire was created.

### Statistical analyses

The structural validity of the questionnaire was verified using factor analysis to analyze the data; the factor analysis was conducted with the freeware statistical package Jamovi (V2.25). The data were randomly divided into two groups: exploratory factor analysis (EFA) was performed on the data from one group (*n* = 176), and confirmatory factor analysis (CFA) was performed on the data from the other group (*n* = 177). The degree of fit of the CFA model was assessed by common statistical parameters, including the chi-square (χ^2^) test, the standardized root mean residual (SRMR), the root mean square error of approximation (RMSEA), the Tucker-Lewis Index (TLI), and the Comparative Fit Index (CFI). The reliability of the questionnaire was analyzed by test-retest reliability, split-half reliability, alternate reliability, and the internal consistency coefficient. All other statistical computations, including bivariate Spearman’s correlations and group comparisons were conducted using the SPSS (V25) statistical software package.

### Ethics and participant’s consent

This study has been approved by the Ethics Committee of the Affiliated Hospital of Guilin Medical University [Approval Number: QTLL202157]. During the evaluation process, the subjects of the study gave their informed consent and signed consent forms on site. The participation of subjects was based on the principle of “proportional universalism” and covered vulnerable groups rather than being targeted [15].

## Results

### Sociodemographic characteristics of the samples

A total of 374 questionnaires were distributed to adults, and all 374 of them were returned, resulting in an effective recovery rate of 100%. Excluding questionnaires with missing answers and repetitive answers, 353 valid questionnaires were obtained, for an effective rate of 94.39%. Two-thirds of the participants were female (63.5%, *n* = 353), and the mean age of the sample was somewhat over 55 years (M = 55.75; SD = 10.10), ranging from 35 to 76 years. The largest portion of the sample consisted of respondents with a college or a higher level of education (31.2%), followed by senior high-school graduates (44.7%), graduates of junior middle-school (17.3%), and participants with a primary education (6.8%). The occupations of the participants were mainly retirees (45.3%), technicians (24.6%), administrators (8.5%), farmers (3.1%), and others (18.4%).

### Cultural adjustment results

After three rounds of evaluation and cultural background debugging for language habits, cultural background, content relevance, etc., the team added two items about smoking and sleep, which were based on items in the original knowledge dimension of the ABCD; the two items added to the knowledge dimension were item 9 (“People who smoke are at risk of having a heart attack or stroke”) and item 10 (Having enough sleep (7–8 hours per day) will help you lower your risk of having a heart attack or stroke”). In contrast, item 7 (“HDL refers to ‘good’ cholesterol, and LDL refers to ‘bad’ cholesterol”) was deleted because it appeared to be too specialized, as nearly half of the people (49.29% (*n* = 173) who completed the pre- test failed to respond to the item. Hence, there were finally nine items in the knowledge dimension. Because Chinese residents have different living habits than foreign residents, Chinese residents found it difficult to understand terms such as gardening and moderate intensity exercise. Therefore, the relevant content of items 2, 3, 6, and 22 were interpreted. For example, the translation of “gardening” in item 2 was interpreted as “digging to plant vegetables or flowers.” For item 3, “moderate intensity exercise” was defined as “running or activities at 60% to 70% of maximum heart rate, where maximum heart rate (times /min)=220-age.” Due to the different drinking habits of Chinese residents, item 6 “drinking high levels of alcohol” was translated as “excessive drinking” (daily alcohol intake > 24 g; note: The amount of alcohol intake was calculated as alcohol content (% v/v) × drinking amount (mL)/100 × 0.8 of the bottle). Weight is usually calculated by kilogram or jin in China, whereas, it is usually based on portions in foreign countries; therefore, “five portions of fruit and vegetables” were annotated as “400 g or 8 liang.”

### The validity of the ABCD

#### Content validity

Seven CVD experts were invited to evaluate the Content Validity Index (CVI) of the ABCD, which was assessed with the CVI at the Item level (I-CVI), the Scale-level Content Validity Index/Universal Agreement Validity Index (S-CVI/UA), and the Scale-level Content Validity Index Average (S-CVI/Ave). A 4-level scoring method was adopted, with scores ranging from 1 (irrelevant) to 4 (very relevant). The I-CVI was 3 or 4 points for each item divided by the total number of experts; the S-CVI/UA was 3 or 4 points for all items, divided by the total number of experts; and the S-CVI/Ave was the average of the I-CVI for all items. The values of the I-CVI, S-CVI/UA, and S-CVI/Ave were .86–1.00, .82, and .97, which indicate good content validity.

#### Construct validity

**Fig. 1:**
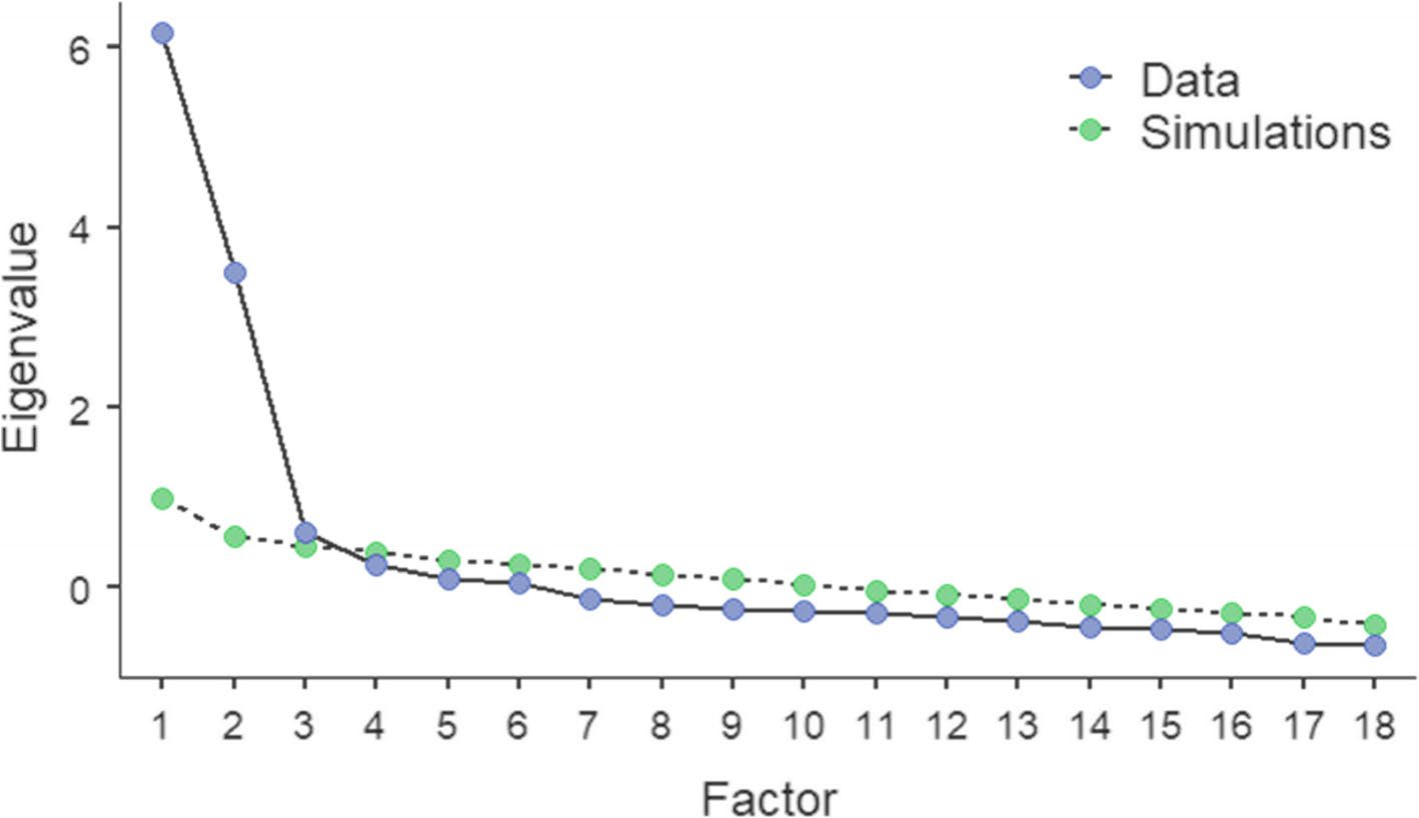
Scree Plot of the EFA

The sample data was suitable for factor analysis based on the Kaiser-Meyer-Olkin (KMO) measure and Bartlett’s test of sphericity. In this study, the KMO of .86 and Bartlett’s χ^2^ value of 2453.0 (*P* < 0.01) met the conditions for EFA, and the cumulative variance contribution rate was 62.84%. A sufficient number of factors were determined from the Scree Plot and a parallel analysis (PA). In PA, the data can be used to generate a certain number of simulated datasets, so the factors whose eigenvalues were greater than 1.00 and higher than the threshold value extracted to obtain three factors, were compared with the original ABCD factors, and found to be the same (Fig. 1). The EFA was conducted by using the maximum variance method to evaluate the item results, which showed that all the items were close to .40, as shown in Table 1.

**Table 1:** Factor loadings of the EFA

	Factor			
Item	1	2	3	Uniqueness
Perceived Risk 6	0.89			0.19
Perceived Risk 5	0.89			0.19
Perceived Risk 4	0.86			0.24
Perceived Risk 3	0.85			0.22
Perceived Risk 2	0.84			0.27
Perceived Risk 1	0.77			0.35
Perceived Risk 8	0.76			0.39
Perceived Risk 7	0.63			0.59
Healthy Eating Intentions 1		0.76	0.33	0.40
Healthy Eating Intentions 2		0.71		0.36
Perceived Benefits 6		0.71		0.43
Perceived Benefits 7		0.67	0.35	0.39
Healthy Eating Intentions 3		0.50		0.71
Perceived Benefits 5		0.39		0.83
Perceived Benefits 1			0.88	0.14
Perceived Benefits 2			0.87	0.13
Perceived Benefits 3		0.46	0.69	0.28
Perceived Benefits 4		0.45	0.54	0.48

The “Minimum residual” extraction method was used in combination with “Varimax” rotation; the hidden loadings were below 0.3

The CFA was used to test the ABCD’s structural validity further by determining the degree to which it fit the EFA model. The results showed that the model fit was acceptable (χ^2^ = 698.79, df = 243, χ^2^/*df* = 2.87, *P* < 0.001; SRMR = 0.06; RMSEA = 0.05; CFI = 0.96; and TLI = 0.94) as shown in Table 2.

**Table 2:** Factor loadings of the CFA

			95% Confidence Interval			
Factor	Indicator	Stand. Estimate	Lower	Upper	***Z***	***p***
Factor 1	Perceived Risk 1	0.87	0.73	0.96	14.65	< 0.001
	Perceived Risk 2	0.82	0.70	0.94	13.31	< 0.001
	Perceived Risk 3	0.80	0.69	0.94	12.96	< 0.001
	Perceived Risk 4	0.81	0.64	0.86	13.13	< 0.001
	Perceived Risk 5	0.96	0.90	1.136	17.58	< 0.001
	Perceived Risk 6	0.74	0.61	0.86	11.49	< 0.001
	Perceived Risk 7	0.89	0.81	1.05	15.26	< 0.001
	Perceived Risk 8	0.97	0.89	1.12	17.74	< 0.001
Factor 2	Perceived Benefits 1	0.81	0.53	0.72	13.07	< 0.001
	Perceived Benefits 2	0.95	0.62	0.78	16.99	< 0.001
	Perceived Benefits 3	0.85	0.57	0.75	14.13	< 0.001
	Perceived Benefits 4	0.78	0.54	0.75	12.37	< 0.001
	Perceived Benefits 5	0.91	0.62	0.80	15.74	< 0.001
	Perceived Benefits 6	0.91	0.55	0.71	15.79	< 0.001
	Perceived Benefits 7	0.70	0.46	0.67	10.62	< 0.001
Factor 3	Healthy Eating Intentions 1	1.00	0.93	1.15	18.84	< 0.001
	Healthy Eating Intentions 2	0.83	0.74	0.98	13.74	< 0.001
	Healthy Eating Intentions 3	0.98	0.92	1.14	18.12	< 0.001

### The reliability of the questionnaire

Cronbach’s α is commonly used as the internal consistency coefficient of a questionnaire. Our studies have shown that the Cronbach’s α of the entire questionnaire was .86, and it was .65, .90, .88, and .78 for each of the four dimensions. Split-half reliability was calculated by the odd and even grouping method. Spearman’s correlation was used to analyze the two halves of the data. The results showed that the correlation of the entire questionnaire was .67, and the correlation of each dimension was .63, .79, .78, and .62. The test-retest reliability of the questionnaire was based on the correlation between the pretest and retest data, using Pearson’s correlation coefficient, to test the repeatability of the results. Three weeks after the 40 participants who took the pre-test of the ABCD, completed a post-test of it; the test-retest reliability of the questionnaire was .97 (*P* < 0.05). The relationship of the questionnaire data with the demographic characteristics of the Chinses sample are presented in Table 3.

**Table 3:** Group comparisons of the questionnaire

Characteristics	n(%)	ABCD Risk Questionnaire				
		Mean (SD)				
		Knowledge	Risk	Benefits	Eating	Total
**Total**	**353**	**7.40 (1.69)**	**15.98 (7.63)**	**20.24 (4.87)**	**7.75 (2.78)**	**51.38 (11.89)**
**Gender**						
Female	224 (63.46)	7.39 (1.69)	16.04 (6.80)	19.80 (4.87)	7.58 (3.06)	51.69 (11.97)
Male	129 (36.54)	7.41 (1.70)	15.94 (8.09)	20.50 (4.86)	7.85 (2.61)	50.86 (11.77)
*P*		0.910	0.902	0.198	0.393	0.528
**Educational level**						
Primary degree or below	24 (6.80)	7.66 (1.49)	16.04 (7.51)	19.95 (5.13)	7.58 (2.04)	51.25 (11.62)
Junior middle-school degree	61 (17.28)	7.27 (1.75)	14.81 (7.86)	20.45 (4.57)	7.91 (2,71)	50.47 (12.85)
Senior high-school degree	158 (44.76)	7.18 (1.90)	14.30 (6.95)	20.20 (4.84)	7.65 (2.94)	49.34 (11.02)
College degree or higher	110 (31.16)	7.73 (1.31)	19.01 (7.65)	20.25 (5.06)	7.85 (2.74)	54.86 (11.98)
*P*		0.034^a^	<0.001^a^	0.976	0.884	0.002^a^
**Employment status**						
Retirees	160 (45.33)	7.18 (1.85)	15.89 (8.31)	20.06 (5.54)	7.73 (2.89)	50.87 (13.09)
Technicians	87 (24.65)	7.36 (1.62)	16.68 (5.97)	19.82 (4.24)	7.71 (2.66)	51.59 (9.82)
Administrators	30 (8.50)	8.23 (0.81)	16.03 (7.84)	21.06 (4.63)	7.80 (2.38)	53.13 (11.01)
Farmers	11 (3.12)	7.72 (1.19)	14.90 (5.35)	20.45 (1.86)	8.09 (1.30)	51.18 (5.89)
Others	65 (18.41)	7.56 (1.67)	15.40 (8.22)	20.84 (4.32)	7.78(3.06)	52.60 (12.62)
*P*		<0.001^a^	0.756	0.607	0.995	0.904
**Residential location**						
Urban	314 (88.95)	7.36 (1.76)	15.94 (7.77)	20.37 (4.79)	7.76 (2.81)	51.45 (12.22)
Suburban	22 (6.23)	7.36 (1.00)	15.45 (6.38)	17.59 (6.11)	7.27 (2.88)	47.68 (8.35)
Rural	17 (4.82)	8.11 (0.92)	17.23 (6.67)	21.29 (3.58)	8.23 (2.13)	54.88 (8.07)
*P*		0.016^a^	0.754	0.080	0.559	0.164
**Annual household income (yuan, RMB)**						
< 50,000,00	121 (34.28)	7.33 (1.75)	15.48 (7.19)	19.55 (4.68)	7.37 (2.86)	49.74 (11.48)
50,000,00-100,000,00	131 (37.11)	7.50 (1.79)	16.01 (8.00)	20.67 (4.72)	7.77 (2.63)	51.96 (12.14)
>100,000,00	101 (28.61)	7.36 (1.50)	16.52 (7.69)	20.52 (5.22)	8.18 (2.83)	52.60 (11.94)
*P*		0.696	0.802	0.152	0.093	0.159
**Smoking status**						
Smoker	155 (43.91)	6.33 (1.92)	12.98 (8.00)	19.47 (5.09)	7.33 (3.11)	46.13 (12.35)
Non-smokers	198 (56.09)	8.24 (0.81)	18.32 (6.44)	20.84 (4.61)	8.08 (2.45)	55.50 (9.73)
*P*		<0.001^a^	<0.001^a^	0.009^a^	0.014^a^	<0.001^a^
**BMI(kg/m**^**2**^**)**						
< 18.5	38 (10.76)	6.65 (2.17)	4.81(5.50)	18.50 (6.60)	6.26 (3.53)	36.23 (11.43)
18.5–23.9	153 (43.34)	8.42 (0.64)	17.88(6.83)	20.98 (4.61)	8.16 (2.50)	55.45 (10.22)
≥24	162 (45.89)	6.61 (1.75)	16.80(6.55)	19.95 (4.51)	7.72 (2.73)	51.09 (10.50)
*P*		<0.001^a^	<0.001^a^	0.033^a^	0.007^a^	<0.001^a^

Body Mass Index (BMI) is a person’s weight in kilograms (or pounds) divided by the square of height in meters (or feet). ^a^ The significance level of the mean difference is .05

## Discussion

The research team adopted the Chinese ABCD and conducted an on-site survey of Chinese adults to verify its psychometric properties, including its content validity and structural validity. Content validity refers to the accuracy of the item content to achieve the expected measurement results (I-CVI ≥ 0.78, S-CVI/UA ≥ 0.8, and S-CVI/Ave ≥ 0.9) [13]. In this study, the I-CVI was .86–1.00, the S-CVI/UA was .82, and the S-CVI/Ave was .97, indicating that the content validity of Chinese ABCD was good. Structural validity reflects the degree of integration between the ABCD’s structure and the theory or framework on which it is based, which requires item loadings that are greater than .40 and a cumulative variance contribution rate not less than 50%. On the whole, all the measurement items had a significance level of *P* < 0.001, and the standardized loadings were all greater than .70 in the EFA results of this study, indicating that there was good correspondence between the factors and the measurement items, and the aggregation validity was good. In addition, the SRMR was close to .08 and the RMSEA was below .06, as required, whereas the TLI and CFI were over .90, indicating a good fit [16]. The factor analysis results confirmed the structural validity of the questionnaire, which was consistent with the results of Martos et al. [8].

Reliability refers to the degree of consistency of the results of a questionnaire across different times, investigators, and scenarios, and it is mainly evaluated by internal consistency/internal reliability, split-half reliability, and test-retest reliability. A Cronbach’s α greater than .70 indicates that a scale’s internal consistency/internal reliability is acceptable, with .65–.70 indicating it is generally acceptable, with.70–.08 indicating it is good, and .80–.90 indicating it is outstanding [17]. The Cronbach’s α of the knowledge dimension of the questionnaire in this study was .65, which was lower than the alpha for translations of the ABCD into Dutch (α = .75) [9], and higher than its translation into Hungarian (α = .50) [8]. This low Cronbach’s α may be due to the specialized knowledge included in the original ABCD questionnaire. Therefore, when the questionnaire is translated into other national languages, it will be translated in accordance with the local language, so that respondents can easily understand it. However, the data for the knowledge items were all within the acceptable range, meaning that the items contributed sufficiently to the overall knowledge score.

Split-half reliability measures the homogeneity of a scale by dividing its items into two parts and calculating the correlation between the two parts. The split-half reliability of a scale is very good if it is over .60, and it was better that that for our version of the ABCD. Retest reliability is an index to evaluate the stability of the scale. For our sample, a large number of correlations showed good stability, and the test-retest reliability of the questionnaire was better than the criterion correlation of .78. Our study, which was conducted in the same hospital, yielded a test-retest reliability for the ABCD of .97, which indicates very high stability.

As for the perception of cardiovascular disease risk, unlike the results of Martos et al. [8], the measures of smoking and Body Mass Index (BMI) were significantly correlated with risk perception, which may be related to the national cultural environment and dietary habits. In China, where tobacco consumption is the highest in the world, smoking has a great impact on people’ s health and it is a well-known risk factor for CVD. Chinese people have a dietary habit that consists of a rich food at dinner, and not exercising after meals [18], which has lead to an increased BMI, but their awareness of the association of the risk for cardiovascular disease with a higher BMI is inadequate. The results of this study showed that people with a high level of education had greater awareness of cardiovascular risks, suggesting that we need to attend to people with lower educational levels in health education in the future. Later studies should pay more attention to these associations and provide targeted individualized education.

### Limitations

Although the methodology used to translate the questionnaire was reasonable, the current research has some limitations. For example, the Jamovi software we used in the study only met the requirements of first-order CFA, and it failed to modify the model. In addition, the study’s sample was obtained by convenience sampling and consisted mostly of urban residents who participated in the early risk screening program of the National Center for Cardiovascular Diseases. Therefore, this may have resulted in self-selection bias. Further assessments of the ABCD should use other methods to provide a more balanced sample.

## Conclusion

In summary, the English version of the ABCD questionnaire was translated into Chinese in this study following strict methodological standards for translating measurement tools, and we added content to measure smoking- and sleep-related knowledge. After deleting two items with low response rates and high repetition rates, the Chinese version of the ABCD we created has 27 items. The reliability and validity of the ABCD was only tested with adults, so other studies are needed with younger samples. The Chinese version of ABCD maintained the content and semantic equivalence of the English version as much as possible, and the Chinese version has good reliability and validity. However, its split-half reliability is low, and the sample size should be increased in subsequent studies. The Chinese version of ABCD provides a reliable tool for assessing the public’s health beliefs about the risk of CVDs, and it provides a self-assessment tool to enhance the public’s awareness of early prevention of CVDs.

## Supplementary Information

**Additional file 1.**

## Data Availability

The datasets generated and analyzed during the current study are not publicly available due to the requirements of the National Cardiovascular Center of China for permitting access to foreign researchers, but they are available from the corresponding author upon a reasonable request.
