# Supplementary material for: Translation and validation of the Chinese ABCD risk questionnaire to evaluate adults’ awareness and knowledge of the risks of cardiovascular diseases
Source: BMC Public Health. 2022 Sep 3;22:1671. doi: 10.1186/s12889-022-14101-z (PMC9441105; doi:10.1186/s12889-022-14101-z)
Supplement: Supplementary file 1 — Additional file 1. [file 12889_2022_14101_MOESM1_ESM.docx]

**The ABCD (Attitudes and Beliefs about Cardiovascular Disease ) Risk Questionnaire and scoring guide**

| **Scale** | **Items** | **Coding** |
| --- | --- | --- |
| **Knowledge**  Higher sum score=more  Knowledgeable  /more correct  about having a  heart attack or  stroke | 1. One of the main causes of heart attack and stroke is stress. | Correct Answers:Q1-T  Q2-T  Q3-T  Q4-T  Q5-T  Q6-T  Q7-F  Q8-T  Q9-T  T=True  F=False  Correct:  Score=1,  Incorrect or  Don’t Know:  Score=0 |
|  | 2. Walking and gardening are considered types of exercise that  can lower the risk of having a heart attack or stroke. |  |
|  | 3. .Moderately intense activity of 2 ½ hours a week will reduce your chances of having a heart attack or stroke. |  |
|  | 4. People who have diabetes are at higher risk of having a heart attack or stroke. |  |
|  | 5. Managing your stress levels will help you to manage your  blood pressure. |  |
|  | 6. Drinking high levels of alcohol can increase your cholesterol and triglyceride levels. |  |
|  | 7. A family history of heart disease is not a risk factor for high blood pressure. |  |
|  | 8. People who smoke are at risk of having a heart attack or  stroke. |  |
|  | 9. Having enough sleep (7-8 hours per day) will help you lower your risk of having a heart attack or stroke”. |  |
| **Perceived Risk ofHeart**  **Attack/Stroke**  Higher sum score =higher  perception of risk of having a heart attack or stroke | 10. I feel I will suffer from a heart attack or stroke sometime  during my life. | 1=Strongly  disagree;  2=disagree;  3=agree;  4=strongly  agree;  N/A=0 |
|  | 11. It is likely that I will suffer from a heart attack or stroke in the future. |  |
|  | 12. It is likely that I will have a heart attack or stroke some  time during my life. |  |
|  | 13. There is a good chance I will experience a heart attack or  stroke in the next 10 years. |  |
|  | 14. My chances of suffering from a heart attack or stroke in the next 10 years are great. |  |
|  | 15. It is likely I will have a heart attack or stroke because of  my past and/or present behaviours. |  |
|  | 16. I am not worried that I might have a heart attack or stroke. | Reverse coded  4=Strongly  disagree;  3=disagree;  2=agree;  1=strongly agree;  N/A=0 |
|  | 17. I am concerned about the likelihood of having a heart attack or stroke in the near future. | 1=Strongly  disagree;  2=disagree;  3=agree;  4=strongly agree;  N/A=0 |
| **Perceived**  **Benefits and**  **Intentions to**  **Change**  Higher average  score=Higher  perceived  benefits of diet  and exercise and higher perceived  readiness for  change in regards to exercise  behaviour. | 18. I am thinking about exercising at least 2½ hours a week. | 1=Strongly  disagree;  2=disagree;  3=agree;  4=strongly agree;  N/A=0 |
|  | 19. I intend or want to exercise at least 2½ hours a week. |  |
|  | 20. When I exercise for at least 2½ hours a week I am doing  something good for the health of my heart. |  |
|  | 21. I am confident that I can maintain a healthy weight by  exercising at least 2½ hours a week within the next two months. |  |
|  | 22. I am not thinking about exercising for 2 ½ hours a week. | Reverse coded  4=Strongly  disagree;  3=disagree;  2=agree;  1=strongly agree;  N/A=0 |
|  | 23. When I eat at least five portions of fruit and vegetables a  day I am doing something good for the health of my heart. | 1=Strongly  disagree;  2=disagree;  3=agree;  4=strongly agree;  N/A=0 |
|  | 24. Increasing my exercise to at least 2½ hours a week will  decrease my chances of having a heart attack or stroke. |  |
| **Healthy Eating**  **Intentions**  Higher average  score=Higher  perceived  readiness for  change with  regard to health  dietary behaviour | 25. I am confident that I can eat at least five portions of fruit  and vegetables per day within the next two months. | 1=Strongly  disagree;  2=disagree;  3=agree;  4=strongly agree;  N/A=0 |
|  | 26. I am thinking about eating at least five portions of fruit and vegetables a day. |  |
|  | 27. I am not thinking about eating at least five portions of fruit and vegetables a day. | Reverse coded  4=Strongly  disagree;  3=disagree;  2=agree;  1=strongly agree;  N/A=0 |
